# Benchmarking inference methods for water quality monitoring and status classification

**DOI:** 10.1007/s10661-020-8223-4

**Published:** 2020-04-02

**Authors:** Hoseung Jung, Cornelius Senf, Philip Jordan, Tobias Krueger

**Affiliations:** 1 Integrative Research Institute on Transformations of Human-Environment Systems, Humboldt-Universität zu Berlin, 10099 Berlin, Germany; 2 School of Geography and Environmental Sciences, Ulster University, Coleraine, BT52 1SA UK

**Keywords:** Water Framework Directive, Ecological status classification, Bayesian uncertainty quantification, Second-order uncertainty, High-frequency monitoring, Phosphorus

## Abstract

River water quality monitoring at limited temporal resolution can lead to imprecise and inaccurate classification of physicochemical status due to sampling error. Bayesian inference allows for the quantification of this uncertainty, which can assist decision-making. However, implicit assumptions of Bayesian methods can cause further uncertainty in the uncertainty quantification, so-called second-order uncertainty. In this study, and for the first time, we rigorously assessed this second-order uncertainty for inference of common water quality statistics (mean and 95th percentile) based on sub-sampling high-frequency (hourly) total reactive phosphorus (TRP) concentration data from three watersheds. The statistics were inferred with the low-resolution sub-samples using the Bayesian lognormal distribution and bootstrap, frequentist *t* test, and face-value approach and were compared with those of the high-frequency data as benchmarks. The *t* test exhibited a high risk of bias in estimating the water quality statistics of interest and corresponding physicochemical status (up to 99% of sub-samples). The Bayesian lognormal model provided a good fit to the high-frequency TRP concentration data and the least biased classification of physicochemical status (< 5% of sub-samples). Our results suggest wide applicability of Bayesian inference for water quality status classification, a new approach for regulatory practice that provides uncertainty information about water quality monitoring and regulatory classification with reduced bias compared to frequentist approaches. Furthermore, the study elucidates sizeable second-order uncertainty due to the choice of statistical model, which could be quantified based on the high-frequency data.

## Introduction

Global water quality has deteriorated in recent decades due to increased pollution from different sources (Seitzinger et al. 2010). In particular, nutrient run-off from point and diffuse sources into surface and ground waterbodies increased problems such as eutrophication and anoxic conditions and impeded water use (Smith 2003; Vörösmarty et al. 2010). Water quality monitoring is an important tool in analysing temporal and spatial trends of water quality, identifying emerging environmental issues, planning measures to mitigate pollution, and evaluating the effectiveness of such measures (Bradley et al. 2015). In the European Union (EU), the Water Framework Directive (WFD) stipulates targets of improvement of the water environment and outlines water management measures that member states should implement (EU 2000). According to the WFD, physicochemical quality of waterbodies should be monitored regularly and river basin management plans produced accordingly. The physicochemical status for a water quality variable is classified with statistics, such as the mean or percentiles, on a predefined classification scale of the variable (EU 2003b; Collins and Voulvoulis 2014). The Directive only specifies a minimum frequency of monitoring, and hence, water quality is usually monitored at limited frequency, which is typical also for other parts of the world (Alexander et al. 1998; EU 2009).

Low-frequency water quality monitoring can cause false grading of the physicochemical status of rivers due to temporal sampling error (Carstensen 2007; Skeffington et al. 2015; Krueger 2017), especially in environments where sources, pollution delivery, and dilution can be very dynamic between low and high flows (Jordan et al. 2005; Jordan et al. 2012). Failure to account for the uncertainty in status determination may impede evaluation of water quality management and policies. Under-grading the status would lead to false incompliance with the WFD, making investments into measures inefficient. Over-grading, on the other hand, can make decision-makers overly optimistic. The risk of false grading is widely challenging for water management as the physicochemical status of waterbodies is assessed in similar ways across the EU and in countries beyond the European continent (e.g. Buck et al. 2000; Zhao et al. 2016).

Uncertainty quantification of water quality data and modelling characterises potential errors stemming from different sources including sampling, analysis, and the complexity of the system of interest (McMillan et al. 2012; Jia et al. 2018; Tasdighi et al. 2018). It can provide decision-makers with critical information on the magnitude of uncertainty to guide management measures and monitoring programs (e.g. Vandenberghe et al. 2007; Brouwer and De Blois 2008). One approach to uncertainty quantification is Bayesian statistics, which computes probability distributions of the statistics of interest (McBride and Ellis 2001; Smith et al. 2001; Borsuk et al. 2002). There have been increasing attempts in recent years to evaluate water quality using Bayesian inference and modelling (e.g. Liang et al. 2016; Xie et al. 2019; Worrall et al. 2020). The Bayesian parametric approach quantifies the uncertainty in the statistics by assuming a particular shape of a population distribution; but the resultant statistics can be biased if the assumption is inappropriate (Krueger 2017). A non-parametric alternative is the bootstrap, which does not rely on any distributional assumption, yet rests on the premise that datasets measured at limited resolution are sufficiently representative of their populations (Fortin et al. 1997; Hirsch et al. 2015; Krueger 2017). A second non-parametric alternative is the more general multinomial model, which – however – requires difficult prior judgements to be made about the data that the low-resolution sampling had missed (Krueger 2017). Choosing a distribution and making other implicit assumptions about the uncertainty in the data shifts uncertainty to so-called second-order uncertainty. This type of uncertainty has drawn attention recently in communities using Bayesian statistics (Hosni 2014; Kaplan and Ivanovska 2018) of climate scientists (Steel 2016) and hydrochemists (Cooper et al. 2014). In practice, it is difficult to assess second-order uncertainty because the population distribution and its statistics are unknown. However, water quality measured at high frequency can provide accurate information on the population distribution and hence can be used as a benchmark to assess otherwise inferred statistics.

In order to assess the second-order uncertainty of competing statistical models, the present study compared the performance of lognormal, gamma, and Weibull distributions; their corresponding bimodal mixtures; and the non-parametric Bayesian bootstrap against high-frequency distributions and statistics uniquely calculated with an hourly dataset of total reactive phosphorus (TRP) concentrations as the benchmark. The performances were compared against those of the classical *t* test and face-value approach used in regulatory practice. The data originated from three river catchment observatories in Ireland that have different hydrological responses to rainfall and thus cover a range of potential monitoring scenarios. These datasets have previously provided important insights into agri-environmental policies (Murphy et al. 2015; Shore et al. 2016) and nutrient hydrological pathway dynamics and seasonality (Jordan et al. 2012; Mellander et al. 2012; Dupas et al. 2017). The specific objectives of the present study were to (1) assess the fitness of different parametric models in reproducing the high-frequency data and (2) compare the performances of these and alternative statistical models in estimating water quality statistics and determining the physicochemical status of the three study rivers.

## Materials and methods

### Study sites

TRP concentrations of three small rural catchments were monitored as part of the Irish Agricultural Catchments Programme (Fealy et al. 2010; Wall et al. 2011). Catchment ‘Arable A’ (11.2 km^2^) is mainly managed for spring barley, and catchments ‘Grassland A’ (7.6 km^2^) and ‘Grassland B’ (12.1 km^2^) support dairy/beef cattle and sheep. Estimated organic P loadings were 9.7 kg ha^−1^ yr^−1^ in Arable A, 23.2 kg ha^−1^ yr^−1^ in Grassland A, and 14.8 kg ha^−1^ yr^−1^ in Grassland B, which were proportional to livestock density (Jordan et al. 2012). Treatment of waste water from rural housing relies on septic tank systems, except in Arable A, where a small package waste water treatment plant with capacity for up to 75 people is operated in addition to the septic tanks. Further characteristics of the catchments have been described in detail in previous studies (Jordan et al. 2012; Mellander et al. 2012).

### Regulatory monitoring programs

The WFD recommends a five-class scheme of ‘high’, ‘good’, ‘moderate’, ‘poor’, and ‘bad’ ecological status. The boundaries of these classes are predefined based on the deviation of waterbodies from estimated undisturbed or reference conditions (EU 2003b). The physicochemical status variables for rivers are biochemical oxygen demand (BOD), dissolved oxygen (DO), pH, temperature, and nutrient concentrations. The overall ecological status of a waterbody is classified using the physicochemical statuses classified for multiple water quality variables together with biological and hydromorphological statuses (EU 2000; Collins and Voulvoulis 2014).

In the Irish regulations, considering the boundaries of ‘moderate’ status and above, statistics calculated from monitoring data are compared with the predefined boundaries (Table 1) to determine the physicochemical status according to each variable (Anonymous 2009). For the classification according to TRP, the mean and the 95th percentile (95%ile) are assessed separately. The status of a river is ‘high’ or ‘good’ when either the mean or the 95%ile lies within the boundaries of these classes. When both of the statistics are in their corresponding ‘moderate’ classes, a river is classified as ‘moderate’ according to TRP.

**Table 1:** Class boundaries of physicochemical status in Irish rivers for TRP concentration

	High	Good	Moderate
Mean (mg P L^−1^)	≤ 0.025	$$ \left\{\begin{array}{c}>0.025\\ {}\le 0.035\end{array}\right. $$	> 0.035
95%ile (mg P L^−1^)	≤ 0.045	$$ \left\{\begin{array}{c}>0.045\\ {}\le 0.075\end{array}\right. $$	> 0.075

The WFD requires member states to monitor the physicochemical status of waterbodies via two types of monitoring programs for different purposes: surveillance monitoring (SM) for the estimation of pollution and identification of sources in large catchments and operational monitoring (OM) for the status assessment of waterbodies ‘at risk of failing to meet their environmental objectives’ (EU 2000). Similar to other EU member states, the Irish Environmental Protection Agency (EPA) measures water quality monthly under SM and five times per year under OM. The water quality data for 3 years are collated to classify the physicochemical status of a river.

### High-frequency water quality monitoring

The TRP concentrations in the three study rivers were rigorously monitored up to three times per hour over 3 years. Water at the outlets of the catchments was sampled and analysed by colorimetry using a fully automated bankside analyser (Hach Sigmatax-Phosphax) (Jordan et al. 2005). The sub-hourly TRP data were aggregated to hourly mean concentrations for data handling. The instruments were calibrated and cleaned through an automated process on a daily basis and were serviced weekly for data transfer and quality management (Jordan et al. 2012). The detection limit of the chemical analysis for TRP was 0.003 mg P L^−1^ (Cassidy and Jordan 2011).

### Frequency distribution analysis

The frequency distribution of the population of a water quality variable such as TRP may be modelled by a parametric probability density function (PDF) making certain assumptions about the shape of the distribution. Provided that the high-frequency data represent the population distribution, the empirical frequency distribution of the data can be used as a benchmark distribution. In this paper, assumed parametric distributions are compared against the benchmark distribution to guide the choice of parametric PDFs when estimating water quality statistics from low-frequency samples. To this end, three unimodal distributions (lognormal, Weibull, and gamma) and three bimodal mixtures of each of these distributions were fitted to the high-frequency data of the three monitoring sites by maximum likelihood, and their log-likelihoods were compared to assess model preference. The unimodal models were chosen based on standard practice (lognormal) and complementary shapes (Weibull and gamma). The mixture models were chosen based on the observed bimodality of some of the high-frequency data. The maximum likelihood parameters of the unimodal and mixture distributions were estimated using the R packages MASS (Venables and Ripley 2002) and mixR (Yu 2018), respectively.

### Sub-sampling experiment

The sampling distribution is the frequency distribution of a certain statistic that is calculated with all possible samples of a given size drawn randomly from a population, which can be approximated numerically by a large number of random draws. A statistic calculated with the high-frequency data approximates the population statistic and is used as a benchmark. The comparison between a sampling distribution and a benchmark statistic quantifies the sampling error (Lahiri 2003). The sampling distributions of the mean and 95%ile were simulated to examine the influence of sampling error on physicochemical status classification. The sampling distributions were simulated by randomly sub-sampling (12 × 3 data points for SM and 5 × 3 data points for OM) the 3-year high-frequency data of each site 10,000 times with replacement and subsequently computing the statistics with each sub-sample. The number of realisations was limited to 10,000 because the sampling distributions showed negligible changes when this number was increased up to 100,000, with Kolmogorov-Smirnov (K-S) statistics ≤ 0.01 for both mean and 95%ile. The sampling was not constrained to certain workdays and hours to simulate the most comprehensive ranges of sampling errors.

The simulated sampling distributions were pooled across the three sites to give information on the errors in the estimated statistics that could be expected in environments similar to those of this study. As the magnitude of sampling errors at each site was proportional to the benchmark statistics, the sampling errors were converted to relative errors as

1 $$ {R}_{\mathrm{e},\mathrm{i}}=\left({X}_{\mathrm{i}}\hbox{--} {X}_{\mathrm{b}}\right)/{X}_{\mathrm{b}}\times 100 $$

where *X*_i_ is the sample statistic of interest calculated with the ith sub-sample, *X*_b_ is the corresponding benchmark statistic, and *R*_e,i_ is the relative error of X_i_.

### Bayesian inference and uncertainty quantification

Bayesian inference yields posterior probability distributions of the statistics of interest, which fully describe their uncertainty conditional on the model. After comparing several parametric models on the high-frequency data (see “Results”), two candidate models emerged for quantifying the uncertainty of inferring the mean and the 95%ile from low-frequency samples, namely, the lognormal distribution and the Bayesian bootstrap. The lognormal model emerged as the best fit among the unimodal parametric distributions to the high-frequency data (see “Results”). The Bayesian bootstrap was selected as a candidate model owing to its flexibility as it does not assume a shape of distribution. Posterior distributions of TRP concentration were inferred using the parametric lognormal model (Gelman et al. 2013) and the Bayesian bootstrap (Rubin 1981; Aitkin 2010) for the 10,000 OM and SM realisations sub-sampled from the high-frequency datasets of the three sites. The posterior parameters of these distributions were sampled by Markov chain Monte Carlo (MCMC), with 1000 realisations (after 1000 burn-in samples). The MCMC sampling was implemented using the Stan software for the parametric distributions and MCMCpack for the Bayesian bootstrap in the R environment (Martin et al. 2011; R Core Team 2017; Stan Development Team 2017) Convergence of the MCMC sampling was tested with a subset of sub-samples using the Gelman-Rubin diagnostic (Gelman et al. 2013). Posterior distributions of the mean and the 95%ile were computed from the posterior parameter distributions. The Bayesian bimodal mixture models, which were appropriate for the high-frequency dataset (see “Results”), had to be abandoned because they were over-parameterised and hence did not converge given the low-resolution sub-samples. Uniform prior distributions were used for the parameters of the distribution models reflecting prior ignorance about the parameters. The mathematical setups of the lognormal model and the bootstrap are detailed in the “Appendix”.

### Analysis of second-order uncertainty

To assess the second-order uncertainty of the lognormal model and the bootstrap in estimating the mean and the 95%ile, the posterior distributions of the two statistics computed with the 10,000 random sub-samples from the high-frequency data were compared against the benchmark statistics. To this end, the hit rate (*H*_R_) and the length of the 95% highest density interval (HDI_95_) were investigated. The hit rate, modified from a suggestion by Schröter et al. (2016), is the proportion of sub-samples from the sub-sampling experiment which include the benchmark statistic within their posterior HDI_95_s:

2 $$ {\mathrm{H}}_{\mathrm{R}}=1/n\cdotp {\sum \limits}_{\mathrm{i}=1}^{\mathrm{n}}{\mathrm{h}}_{\mathrm{i}};{\mathrm{h}}_{\mathrm{i}}=\left\{\begin{array}{c}1,\kern0.5em \\ {}0,\end{array}\right.\kern0.5em {\displaystyle \begin{array}{c}\mathrm{if}\kern0.75em {\mathrm{X}}_{\mathrm{b}}\in \left[{\mathrm{H}\mathrm{D}}_{2.5},{\mathrm{H}\mathrm{D}}_{97.5}\right]\\ {}\mathrm{otherwise}\end{array}} $$

where *n* is the number of sub-samples from the high-frequency dataset (*n* = 10,000), *h*_i_ is the inclusion indicator, and HD_2.5_ and HD_97.5_ are the 2.5th and 97.5th percentiles, respectively. The HDI_95_ was obtained with *R* scripts provided by Kruschke (2010).

Relative mean bias error (RMBE) of each posterior distribution was calculated from the MCMC sample as

3 $$ \mathrm{RMBE}=1/N\cdotp {\sum \limits}_{\mathrm{m}=1}^{\mathrm{N}}\left({\mathrm{X}}_{\mathrm{m}}\hbox{--} {\mathrm{X}}_{\mathrm{b}}\right)/{\mathrm{X}}_{\mathrm{b}}\times 100 $$

where *X*_m_ is the mth MCMC realisation of the statistic of interest (*N* = 1000). A positive RMBE indicates that the posterior distribution generally overestimates the statistic relative to the benchmark and a negative RMBE indicates underestimation.

### Regulatory approaches for physicochemical status classification

The European Commission has outlined various approaches for physicochemical status classification (EU 2003a). One approach is termed ‘face-value approach’, where raw sample statistics are directly used for classification without any consideration of the uncertainty in their estimation. Alternatively, within a frequentist statistical framework, hypotheses that the population statistics are significantly higher or lower than specific class boundaries can be tested. A right-tailed test verifies if a statistic is significantly higher than a class boundary, reducing the risk of overestimating the statistic, i.e. reducing under-grading (‘benefit-of-doubt’ approach) (Carstensen 2007). A left-tailed test examines the opposite hypothesis, reducing the risk of over-grading (‘fail-safe’ approach). In the Irish case, the physicochemical statuses for BOD and nutrients are determined using the right-tailed *t* test in the ‘benefit-of-doubt’ approach. Sample mean and 95%ile are tested whether they are significantly higher than the class boundaries at a confidence level of 99%, and both *t* test results are used to determine the physicochemical status following the scheme described above.

In the Bayesian approach, the uncertainty of classification is quantified directly with the posterior distribution of the mean and the 95%ile, respectively. In keeping with the Irish monitoring program and classification scheme described above, the probability, or confidence, of classifying a river according to BOD or nutrient concentrations as ‘high’, ‘good’ or ‘moderate’ can be quantified as follows:

4 $$ \mathrm{P}\left(\mathrm{Class}=\mathrm{High}\right)=\mathrm{P}\left(\begin{array}{cc}\begin{array}{cc}\begin{array}{cc}{\mathrm{m}}_{\mathrm{a}}& \in \end{array}& \mathrm{High}\end{array}& \cup \end{array}\kern0.5em \begin{array}{cc}{\mathrm{Q}}_{95}& \begin{array}{cc}\in & \mathrm{High}\end{array}\end{array}\right) $$

5 $$ \mathrm{P}\left(\mathrm{Class}=\mathrm{Good}\right)=\mathrm{P}\left(\begin{array}{cc}\begin{array}{cc}{\mathrm{m}}_{\mathrm{a}}& \in \end{array}& \mathrm{Good}\end{array}\kern0.5em \begin{array}{cc}\begin{array}{cc}\begin{array}{cc}\cap & {\mathrm{Q}}_{95}\end{array}& \in \end{array}& \mathrm{Good}\end{array}\right)+\mathrm{P}\left(\begin{array}{cc}\begin{array}{cc}\begin{array}{cc}\begin{array}{cc}\begin{array}{cc}\begin{array}{cc}{\mathrm{m}}_{\mathrm{a}}& \in \end{array}& \mathrm{Good}\end{array}& \cap \end{array}& {\mathrm{Q}}_{95}\end{array}& \in \end{array}& \mathrm{Moderate}\end{array}\right)+\mathrm{P}\left(\begin{array}{cc}\begin{array}{cc}\begin{array}{cc}\begin{array}{cc}\begin{array}{cc}\begin{array}{cc}{\mathrm{m}}_{\mathrm{a}}& \in \end{array}& \mathrm{Moderate}\end{array}& \cap \end{array}& {\mathrm{Q}}_{95}\end{array}& \in \end{array}& \mathrm{Good}\end{array}\right) $$

6 $$ \mathrm{P}\left(\mathrm{Class}=\mathrm{Moderate}\right)=\mathrm{P}\left(\begin{array}{cc}\begin{array}{cc}\begin{array}{cc}\begin{array}{cc}\begin{array}{cc}{\mathrm{m}}_{\mathrm{a}}& \in \end{array}& \mathrm{Moderate}\end{array}& \cap \end{array}& {\mathrm{Q}}_{95}\end{array}& \in \end{array}\kern0.5em \mathrm{Moderate}\right) $$

P(Class = X) indicates the posterior probability that the physicochemical status of a river is in class X. P(m_a_ ∈ X) and P(Q_95_ ∈ X) indicate the posterior probabilities that the mean and the 95%ile, respectively, of a water quality variable are within the boundaries of class X.

## Results

### Frequency distribution analysis

**Fig. 1:**
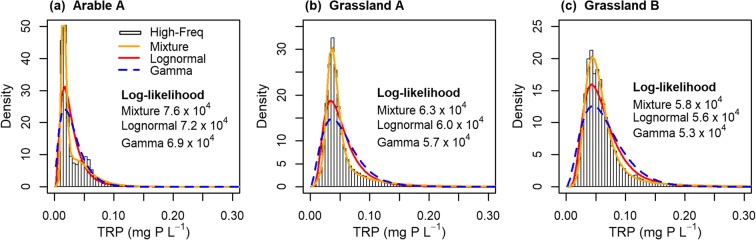
Frequency distributions of hourly TRP concentration from 2011 to 2013 (high-freq), bimodal lognormal mixture (mixture), and unimodal lognormal and gamma distributions fitted to the frequency distributions via maximum likelihood and their log-likelihoods

The high-frequency TRP concentrations at the monitoring sites showed some bimodality with the higher mode possibly representing increased TRP concentrations during storm events, when sediment is flushed from the system (Jordan et al. 2005; Jordan et al. 2007). Comparing the parametric distribution models, the bimodal lognormal mixture showed the best fit to the high-frequency data at all the monitoring sites as indicated by the highest log-likelihoods (5.8 · 10^4^–7.6 · 10^4^, Fig. 1). The bimodal gamma mixture also fitted the data well with log-likelihoods of 5.8 · 10^4^–7.5 · 10^4^ (not shown). The unimodal lognormal distribution yielded fits with log-likelihoods of 5.6 · 10^4^–7.2 · 10^4^, which were the highest among all unimodal distributions tested (Fig. 1). The unimodal gamma and Weibull distributions and the bimodal Weibull mixture (not shown) exhibited lower log-likelihoods of 5.3 · 10^4^–6.9 · 10^4^, 4.9 · 10^4^–6.7 · 10^4^ and 5.6 · 10^4^–6.9 · 10^4^, respectively. Although the bimodal lognormal and gamma mixtures fitted the high-frequency data best, they did not converge on low-frequency sub-samples due to over-parameterisation with respect to the information content in a sub-sample, and hence, the unimodal lognormal model was preferred in the remainder of this study. Among the high-frequency datasets, the lognormal model showed the highest log-likelihood for Arable A (7.2 · 10^4^), followed by Grassland A (6.0 · 10^4^) and B (5.6 · 10^4^).

### Sampling distributions

**Fig. 2:**
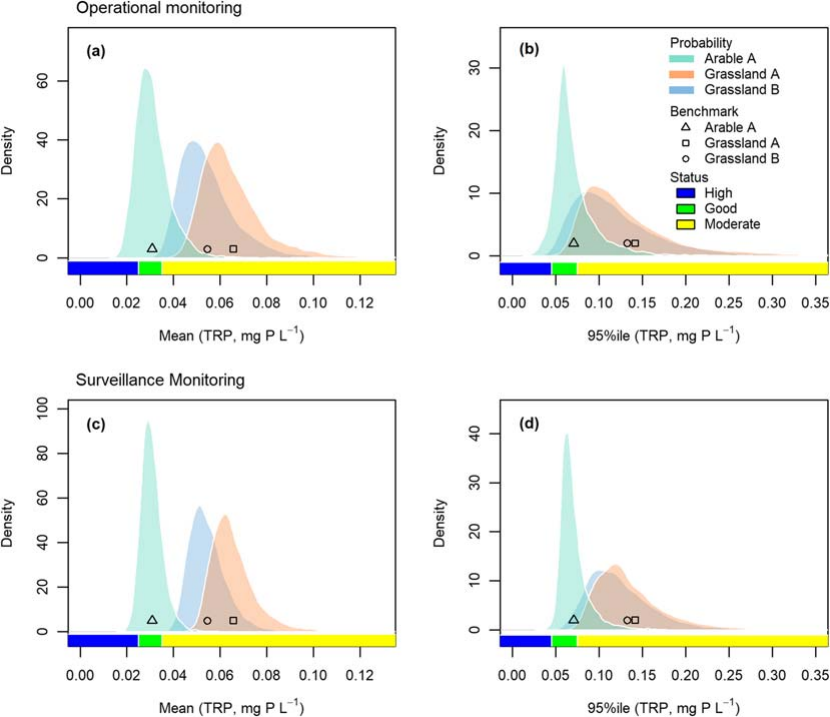
Mean and 95%ile calculated with the high-frequency data (2011–2013) and their sampling distributions simulated by sub-sampling the high-frequency data according to operational (OM; **a** and **b**) and surveillance (SM; **b** and **c**) monitoring schemes

The simulated sampling distributions of the mean and the 95%ile were right skewed, suggesting that the chances of underestimating the statistics due to the sampling error were greater than the chances of overestimating them (Fig. 2). However, the overestimations had a greater range than the underestimations, which were bound below by zero. Comparing the means of the sampling distributions with the benchmark statistics revealed that the sampling was unbiased for the mean and hardly biased for the 95%ile (Table 2). The skewness values of the sampling distributions show that the sampling distributions of the 95%ile were more strongly right skewed than those of the mean and that the sampling distributions became less skewed as the sample size increased from OM (5/year) to SM (12/year). The widths of the HDI_95_s of the sampling distributions from SM were also less variable than those from OM. The HDI_95_s are comparable to or larger than the widths of the ‘good’ classes for the mean and 95%ile of TRP (see Table 1).

**Table 2:** Mean, skewness, and width of 95% highest density interval (HDI_95_) of sampling distributions of mean and 95th percentile simulated under operational monitoring (OM) and surveillance monitoring (SM) scenarios

		Operational monitoring		Surveillance monitoring	
		Mean	95%ile	Mean	95%ile
Mean	Arable A	0.00	− 0.01	0.00	− 0.01
(mg P L^−1^)	Grassland A	0.00	− 0.01	0.00	− 0.01
	Grassland B	0.00	0.00	0.00	0.00
Skewness	Arable A	2.58	3.36	1.61	1.75
(−)	Grassland A	1.58	3.08	1.07	2.47
	Grassland B	1.38	1.9	0.87	1.34
HDI_95_	Arable A	0.05	0.2	0.04	0.14
(mg P L^−1^)	Grassland A	0.03	0.09	0.02	0.06
	Grassland B	0.04	0.18	0.03	0.14

**Fig. 3:**
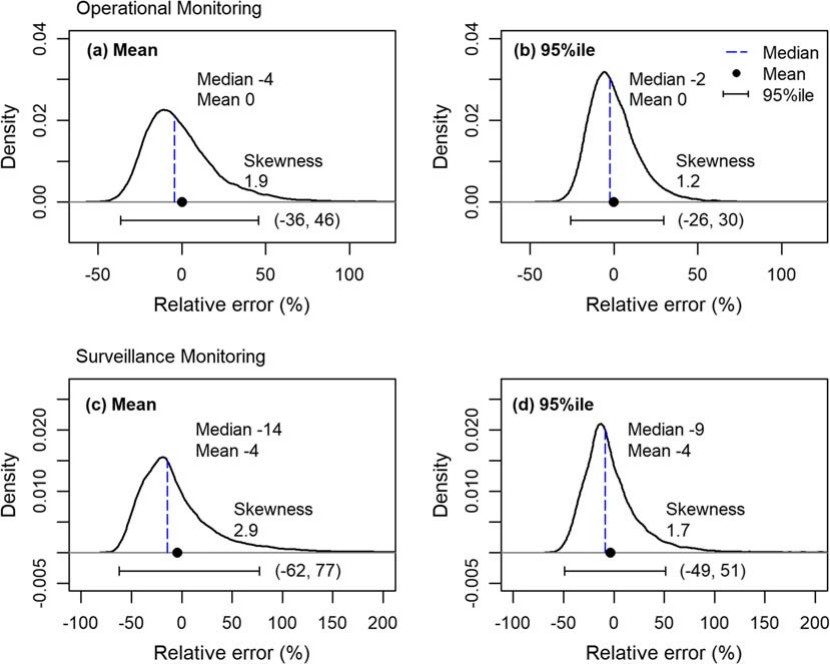
Sampling distributions of relative errors of mean (**a** and **c**) and 95th percentile (**b** and **d**) pooled across study sites simulated under operational monitoring (OM; **a** and **b**) and surveillance monitoring (SM; **c** and **d**) scenarios

The sampling distributions expressed as relative error were pooled across the three sites as a measure of expected sampling error, which verified that the expected sampling error for the mean and 95%ile decreased, showing narrowed HDI_95_s, as the sample size increased (Fig. 3). These results also indicate that the sampling error of the 95%ile was more variable across sub-samples than that of the mean. The pooled relative sampling error showed no bias for the mean and a negative bias for the 95%ile. The expected error distributions were right-skewed, so their medians were negative. As observed with the sampling distributions at each site, the expected sampling error was more right skewed for the 95th percentile than for the mean, and their skewness decreased with the increase in sample size for both statistics.

### Second-order error of Bayesian inference

Given OM sub-samples, the posterior distributions of the lognormal model tended to have wider HDI_95_s than the posterior distributions estimated by the bootstrap, and the lognormal model captured the statistics in the posterior highest density interval more often than the bootstrap, evidenced by greater hit rates (Table 3). The difference between the two methods was especially compelling for the 95%ile. That is, the posterior distributions of the 95%ile estimated by the bootstrap were narrow (HDI_95_s 0.01 mg P L^−1^), and their hit rates were lower than 10%, whereas the posterior distributions estimated by the lognormal model were wider (median HDI_95_s 0.04–0.08 mg P L^−1^) and captured the benchmark statistics for 75–92% of the sub-samples. The estimation of the mean showed much lower RMBEs than that of the 95%ile. The lognormal model biased the estimation of the 95%ile either positively or negatively, while the bootstrap tended to negatively bias the 95%ile across all sites.

**Table 3:** Second-order uncertainty of estimated mean and 95%ile from random low-resolution sub-samples using Bayesian lognormal model and bootstrap

			HR^a^	HDI_95_^b^ (mg P L^−1^)			RMBE ^c^ (%)		
			(%)	2.5%	Median	97.5%	2.5%	Median	97.5%
Operational monitoring (OM)									
Mean	Lognormal	Arable A	91	0.01	0.03	0.07	− 33	4	77
		Grassland A	86	0.01	0.03	0.11	− 29	0	69
		Grassland B	88	0.02	0.04	0.12	− 26	− 1	64
	Bootstrap	Arable A	85	0.01	0.02	0.05	− 35	− 4	55
		Grassland A	79	0.01	0.03	0.09	− 31	− 4	55
		Grassland B	78	0.01	0.03	0.12	− 29	− 5	57
95%ile	Lognormal	Arable A	92	0.03	0.04	0.07	− 43	13	137
		Grassland A	75	0.05	0.07	0.12	− 52	− 11	105
		Grassland B	81	0.06	0.08	0.14	− 42	− 5	122
	Bootstrap	Arable A	9	0.01	0.01	0.01	− 48	− 13	127
		Grassland A	6	0.01	0.01	0.01	− 60	− 16	119
		Grassland B	6	0.01	0.01	0.01	− 52	− 15	164
Surveillance monitoring (SM)									
Mean	Lognormal	Arable A	89	0.01	0.01	0.03	− 25	− 1	33
		Grassland A	84	0.01	0.02	0.04	− 21	− 2	30
		Grassland B	85	0.01	0.02	0.04	− 20	− 3	28
	Bootstrap	Arable A	91	0.01	0.02	0.03	− 25	− 2	34
		Grassland A	87	0.01	0.02	0.06	− 22	− 2	34
		Grassland B	90	0.02	0.03	0.09	− 21	− 3	38
95%ile	Lognormal	Arable A	90	0.03	0.05	0.09	− 31	3	58
		Grassland A	64	0.03	0.06	0.14	− 43	− 15	35
		Grassland B	74	0.03	0.07	0.16	− 34	− 8	49
	Bootstrap	Arable A	19	0.01	0.01	0.02	− 30	− 2	91
		Grassland A	9	0.01	0.02	0.02	− 44	− 3	86
		Grassland B	8	0.01	0.02	0.02	− 39	− 1	117

^a^Hit rate

^b^Length of 95% highest density interval of the posterior distribution

^c^Relative mean bias error

For the HDI_95_ and the RMBE, the median and central 95% across all sub-samples are given

When the sample size was increased from OM to SM, the posterior HDI_95_s narrowed from 0.03–0.04 (median) to 0.01–0.02 mg P L^−1^ (median) for the mean and from 0.04–0.08 (median) to 0.05–0.07 mg P L^−1^ (median) for the 95%ile in case of the lognormal model (Table 3). The hit rates decreased by 2–3 percentage points for the mean and by 2–11 percentage points for the 95%ile. In Arable A, however, the hit rates were still as high as 90% for both statistics with the SM sub-samples. The median RMBE for both statistics became closer to zero in Arable A (mean by 3 percentage points, 95%ile by 10 percentage points) but deviated farther from zero in Grassland A (mean by 2 percentage points, 95%ile by 4 percentage points) and Grassland B (mean by 2 percentage points, 95%ile by 3 percentage points). However, the distributions of RMBE generally narrowed to zero with increasing sample size; e.g. in Arable A from − 33–77% to −25–33% (central 95%) for the mean and from −43–137% to −43–35% (central 95%) for the 95%ile.

The posterior distributions of the mean estimated by the bootstrap tended to be slightly narrower with increased sample size with HDI_95_ for OM extending to 0.12 mg P L^−1^ (97.5th percentile) and for SM to 0.09 mg P L^−1^ (97.5th percentile) (Table 3). However, their hit rates were enhanced by 6–12 percentage points, and the median RMBEs narrowed towards zero by 2 percentage points. The median RMBEs for the 95%ile moved closer to zero by 11–14 percentage points, which improved the hit rates by 2–10 percentage points. However, these posterior distributions were still narrower than those of the lognormal model with much lower hit rates, and their RMBEs varied more widely (e.g. − 30 to 91% (central 95%) for Arable A) than those of the lognormal model (e.g. – 31 to 58% (central 95%) for Arable A). Examples of uncertainties in the mean and 95%ile quantified by the Bayesian lognormal model and bootstrap given unrepresentative sub-samples are provided in Fig. 5 in the Appendix.

### Uncertainty of physicochemical status classification

**Fig. 4:**
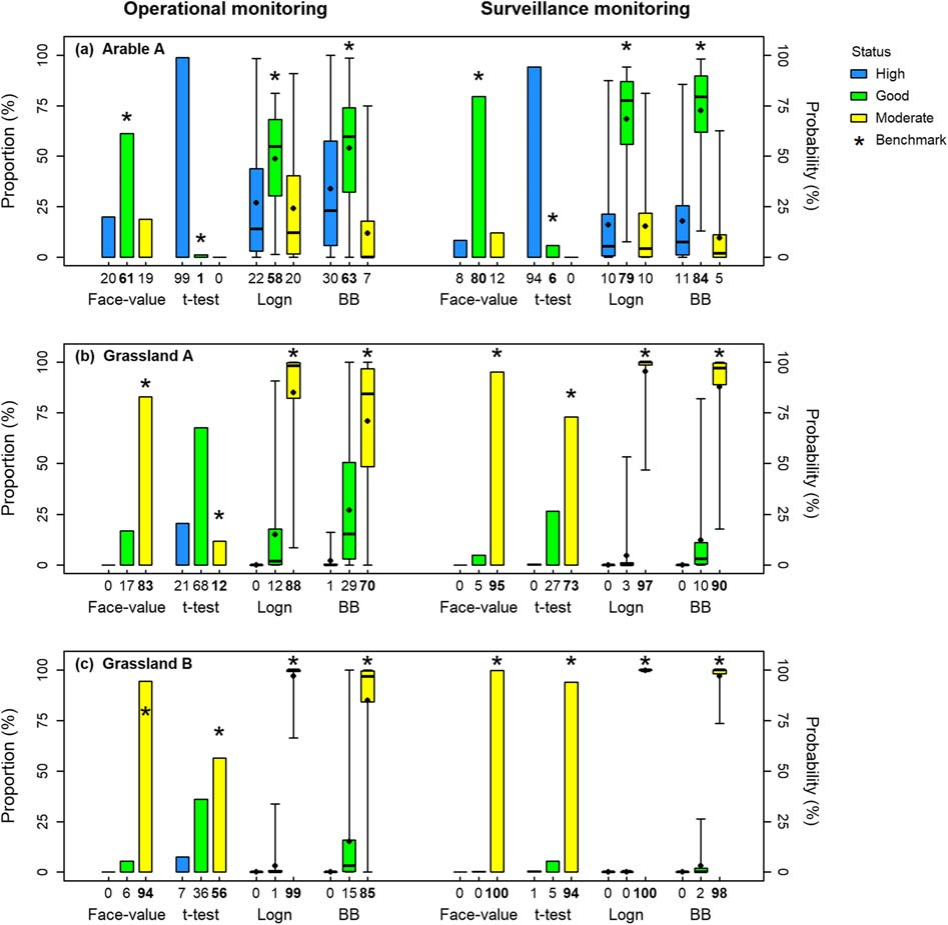
Proportions of physicochemical status classes determined with the face-value approach and the right-tailed *t* test and the confidences of classification estimated with the Bayesian lognormal model (Logn) and the Bayesian bootstrap (BB) given 10,000 operational and surveillance monitoring sub-samples

The performance of the different statistical models in classifying the physicochemical status was assessed by comparing their classification results based on OM and SM sub-samples with the status determined with the high-frequency data, the ‘correct’ benchmark status. The results were summarised as the percentage of sub-samples exhibiting certain behaviour, which is also referred to as the ‘frequency’ of that behaviour in the sub-sampling experiment. The classification was most frequently false in Arable A (39% under OM, 20% under SM) using the face-value approach (Fig. 4), where the benchmark statistics were located in the ‘good’ class, which was narrower than the sampling distributions and near the ‘good/moderate’ boundary (see Fig. 2). The classification was less frequently false in Grassland A (17% under OM, 5% under SM) and Grassland B (6% under OM, 0% under SM) with the same approach, where the ‘true’ classes were ‘moderate’, which is wider than ‘good’. Especially in Grassland B, the benchmark statistics were far from the ‘good/moderate’ boundaries (Fig. 2); hence, the classification was least susceptible to sampling error. The misclassification decreased as TRP was sampled more frequently in the SM scenario.

The right-tailed *t* test frequently over-graded the physicochemical status (43–99% under OM, 6–94% under SM) (Fig. 4). Particularly in Arable A, the physicochemical status was correctly graded for only 1% and 6% of the OM and SM sub-samples, respectively. The confidence intervals of the t-distributions were 0.02 ± 0.02 mg P L^−1^ and 0.01 ± 0.01 mg P L^−1^ when estimated with the OM and SM sub-samples, respectively (not shown), i.e. frequently larger than or comparable to the widths of the ‘good’ classes (0.02 mg P L^−1^ for mean and 0.04 mg P L^−1^ for 95%ile, see Table 1).

**Fig. 5:**
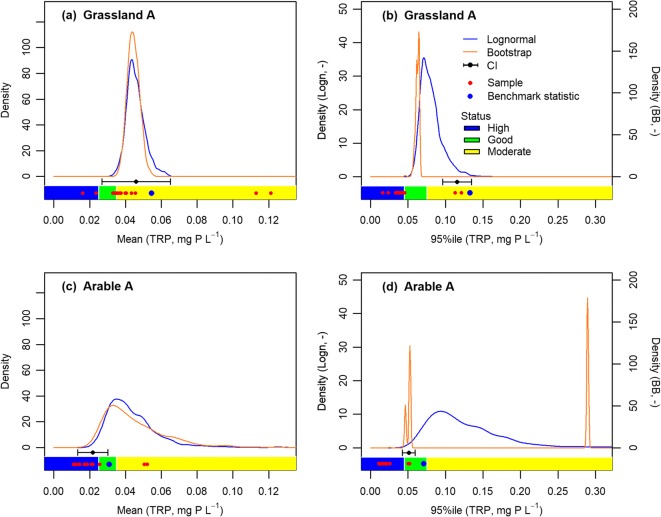
Examples of posterior distributions and confidence intervals (CI) of mean and 95%ile computed using the Bayesian lognormal model (Logn) and Bayesian bootstrap (BB) and the frequentist *t* test, respectively, given lower-biased (**a** and **b**) and upper-biased (**c** and **d**) operational monitoring sub-samples from Grassland A and Arable A, respectively

Using the lognormal model for classification increased the proportions of classes determined correctly by less than 3% compared to the face-value approach in Arable A, where the benchmark statistics were ‘good’. In Grassland A and B, where the benchmark statistics were ‘moderate’, the lognormal model increased the proportions classifying the status correctly by 2–5 percentage points compared to the face-value approach. The model biased the classification less for these sites because even when the sample 95%ile was falsely in the ‘good’ class (face-value approach), large portions of the posterior distributions were located correctly in ‘moderate’ (see an example in Fig. 5b).

When physicochemical status was classified using the bootstrap, the posterior distributions were concentrated around the sub-sampled data points. By definition of percentile, 95% of the data points of a sub-sample are distributed lower than its 95%ile. Consequently, the posterior distributions of the 95%ile estimated by the bootstrap were also located lower than the sample 95%iles (see an example in Fig. 5d). Accordingly, the bootstrap tended to bias the classification towards high classes. That is, the chances of classification as ‘moderate’ decreased, and the chances of classification as ‘high’ or ‘good’ increased compared to the face-value approach, which used the sample 95%iles. This effect enhanced the chances of accurately classifying Arable A, where the correct class was ‘good’, but undermined the accuracy in Grassland A and B where the correct class was ‘moderate’.

The Bayesian inference methods are capable of computing the confidence of classification with the posterior distributions of the statistics using Eqs. (4)–(6). The classification is strongly confident when the data points of a sample are concentrated in a certain class and weakly confident when a sample is widespread as demonstrated in the examples in Fig. 5. Distributions of the confidences of classification calculated with the sub-samples are displayed as box plots in Fig. 4. The medians of the confidences were close to the proportions of classification estimated with the face-value statistics. In the OM scenario in Arable A and Grassland A, classifications were more than 90% confident in pointing to ‘wrong’ classes with more than 5% of the sub-samples. The Bayesian bootstrap tended to show higher confidences for ‘high’ and ‘good’ classes than the lognormal model.

## Discussion

### Errors in Bayesian statistical models

Although the lognormal distribution did not represent the high-frequency data perfectly, it provided the best fit of the unimodal distributions tested here across all monitoring sites as measured by the maximum log-likelihood (Fig. 1). This lognormal distribution of TRP concentration contrasts with Johnes (2007), who described daily total phosphorus concentrations using the gamma distribution, which performed sub-optimally here. Cassidy and Jordan (2011) fitted sub-hourly total phosphorus data with the power-law distribution, which was not tested here because it did not represent low values accurately in the previous study.

In agreement with the good fit of the lognormal distribution to the high-frequency data in this study (Fig. 1), the lognormal model estimated the benchmark statistics (mean and 95%ile) with the lowest relative mean bias errors (RMBEs) and the highest hit rates (Table 3). Given OM sub-samples, whose resolution was extremely low, the model captured the benchmark statistics within its HDI_95_s (Table 3) because its posterior distributions were conservatively wide (see an example in Fig. 5c, d). The RMBE distributions demonstrate that the posterior distributions were subject to sampling error when unrepresentative samples were given, as also exemplified in Fig. 5. With the increased sample size of the SM scenario, the model gained precision but introduced a bias in estimating the benchmark statistics indicated by reduced hit rates and median RMBEs deviating from zero (Table 3). This result supports the finding by Krueger (2017) that parametric models can result in biased inference when samples do not accurately represent the population. Only in Arable A, where the lognormal model fitted the high-frequency data with the highest likelihood (Fig. 1), the hit rates remained high and median RMBEs moved closer to zero with increased sample size, suggesting that the second-order uncertainty at this site was lowest.

The Bayesian bootstrap assumes that an observed sample is representative of the population and its estimation solely depends on that sample (Ebtehaj et al. 2010). The bootstrap does not place (prior) probability on data values missing from the sample (Krueger 2017), and its posterior distributions are discrete (Ebtehaj et al. 2010). Consequently, in the present study, the posterior distributions of the bootstrap were concentrated around the ‘observed’ data points even when the sub-samples were unrepresentative (Fig. 5). The posterior distributions of the bootstrap for the mean had widths and hit rates comparable to those estimated by the lognormal model, because the mean had relatively small sampling errors and was calculated by a continuous equation (see “Appendix”). The 95%ile, however, was calculated in a discrete manner from the results of the bootstrap; thus, its posterior distributions were narrow, discrete, and multimodal. Due to these characteristics of the bootstrap, this method failed to capture the benchmark 95%iles within the HDI_95_s of the posterior distributions in more than 80% of the cases (Table 3). Since the posterior distributions of the bootstrap concentrated narrowly on unrepresentative values, this method can mislead decision-makers with a falsely high level of confidence. The bootstrap performed especially poorly in the estimation of the 95%ile, which is important ecologically since eutrophication is caused by rapid proliferation of algae at high nutrient concentrations (Hilton et al. 2006; Xu et al. 2014). The Bayesian bootstrap combined with a prior distribution of missing data, the multinomial model, can allow inference in ranges with missing data and result in wide posterior distributions (Krueger 2017), which would enable uncertainty quantification without a distributional assumption, yet at the cost of greater imprecision induced by difficult prior choices.

### Comparison of classification methods

Using the face-value approach in this study, there were sizeable chances (maximum 39%) of misclassifying physicochemical status (Fig. 4). The chance of the misclassification was especially high with the OM scenario and when the benchmark statistics were in the narrow ‘good’ class or close to class boundaries, verifying the results of Skeffington et al. (2015). Moreover, the face-value approach does not provide any information about uncertainty and thus is not capable of managing the risk of misclassification (Carstensen 2007). The incapability of the approach to transmit the uncertainty in the classification would exacerbate the policy difficulty induced by the high chance of misclassification.

The right-tailed *t* test, which is a frequentist statistical approach suggested by the European Commission and currently used by the Irish EPA among other agencies in the EU, aims to reduce the risk of falsely diagnosing incompliance with environmental regulations (‘benefit-of-doubt’ approach) (Carstensen 2007). This approach, therefore, yielded overly optimistic classifications in our sub-sampling experiment (Fig. 4), which would leave rivers with poor water quality unmanaged. The left-tailed *t* test, in contrast, would have resulted in pessimistic status classifications (‘fail-safe’ approach), which would arguably be more attuned to the precautionary prescriptions of the WFD (e.g. the ‘one-out-all-out’ principle). However, if a river in ‘good’ conditions or above is pessimistically classified as ‘moderate’ or worse, unnecessary management measures would be executed.

The Bayesian lognormal model, in addition to providing a coherent measure of uncertainty conditional only on the assumed distributional form, either enhanced or decreased the accuracy of classification compared to the face-value approach, depending on the location of the benchmark statistics, but to minimal degrees (0–5%, Fig. 4). The classification results of the lognormal model did not show any clear relation with the directions of the biases indicated by the RMBEs (Table 3), possibly because the biases were small compared to the classification scale. The lognormal model does not necessarily improve the accuracy of classification, but it provides uncertainty information of the classification without bias as with the *t* test method. The uncertainty information can be presented as probabilities of classification or probability distributions of mean and 95%ile (see an example in the Appendix and Fig. 5) and would allow decision-makers to take the possibility of misclassification into account.

The Bayesian bootstrap often biased physicochemical status towards ‘high’ or ‘good’ and away from ‘moderate’ compared to the face-value approach (2–12% of cases) (Fig. 4) due to the over-reliance of its posterior distribution on observed data. This effect of the bootstrap was less pronounced when the sample size was increased from OM to SM because a larger sample is likely to be more representative of the population. Nevertheless, hit rates for the 95%ile remained as low as 8–19%. The tendency of the Bayesian bootstrap to over-grade rivers with over-confidence given low-frequency samples would mislead policies not to manage rivers that in fact need improvement.

### Efficacy of high-frequency water quality data

The high-frequency nutrient concentration data used in this study proved highly beneficial for assessing the second-order uncertainty of the inference methods. The performance of the lognormal model, despite being a popular choice, should be evaluated on further high-frequency datasets from contrasting environments. High-frequency monitoring data should further be used to benchmark the inference methods and statistical models applied to other water quality variables so that the uncertainty in overall WFD physicochemical status classification can be quantified.

High-frequency data are also valuable for constructing empirical distributions of expected sampling error by sub-sampling (sampling distributions). These distributions of expected error may be used in a Bayesian statistical setup as prior distributions for parametric or non-parametric population models in new monitoring situations. The prior information can reduce the risk of false status classification due to low-resolution monitoring by assigning informed probabilities to values not observed in the data at hand. The general applicability of these priors will improve as sampling distributions from diverse environments are pooled as in this study. While our set of nine catchment years is clearly limited, we suggest that a large number of high-frequency datasets across the world be analysed in this way and the resultant sampling distributions pooled to arrive at a more robust prior distribution of sampling error. A large enough dataset may even allow discerning drivers of the variation of sampling uncertainty across different environments and thus a more differentiated selection of priors.

## Conclusions

This study benchmarked inference methods and statistical models in water quality monitoring and status classification by sub-sampling a high-frequency TRP concentration dataset of nine catchment years from Ireland. The high-frequency dataset, used as the benchmark in this study, enabled the assessment of second-order uncertainty caused by the selection of inference methods as well as low-frequency monitoring. The *t* test, which is common regulatory practice in the EU, biased the classification in 44–100% of the cases in the sub-sampling experiment. Bimodal mixture distributions, despite fitting the high-frequency data best, did not converge on the low-resolution sub-samples in this study due to over-parameterisation. The Bayesian lognormal model of the distribution, despite not fitting the high-frequency data perfectly, classified WFD physicochemical status with minimal bias (less than 5% of sub-samples) compared to the face-value approach. This inference method provided reliable uncertainty information to assist policies and thereby outperformed the Bayesian bootstrap, the face-value approach, and the frequentist *t* test. High-frequency nutrient concentration data can guide the selection of inference methods and potentially provide prior information for water quality monitoring. These findings and principles are widely applicable, and further accumulation of high-frequency monitoring data at different sites and for different variables would enable the selection of inference methods and development of efficient priors to expand. Bayesian modelling can be applied to quantify uncertainty of classifying not only the physicochemical status but also the biological status and, consequently, the overall ecological status (Moe et al. 2016; Loga et al. 2018). It is important to discuss further how to apply Bayesian methods to the overall procedure of WFD status classification and which statistical models to use.

## Appendix

### Bayes rule

In Bayes theory, a posterior probability distribution of a parameter vector of interest π(θ|y) is calculated by updating a prior probability distribution π(θ) with a likelihood function L(θ|y):

7 $$ \uppi \left(\uptheta |\mathrm{y}\right)=\mathrm{L}\left(\uptheta |\mathrm{y}\right)\ \uppi \left(\uptheta \right)/\int \mathrm{L}\left(\uptheta |\mathrm{y}\right)\uppi \left(\uptheta \right)\mathrm{d}\uptheta $$

where θ is the parameter vector of interest and y is a vector of observed data, i.e. TRP concentration in this study.

### Parametric estimation with Bayesian lognormal distribution

In a Bayesian parametric model, the data population is assumed to follow a certain shape, whose parameters are estimated via Bayes rule. The likelihood function of the lognormal model is

8 $$ \mathrm{L}\left(\mu, \sigma |y\right)=1/\upsigma \sqrt{2\uppi}\cdotp 1/\mathrm{y}\bullet \exp \left[-1/2\bullet {\left(\log y-\mu \right)}^2/{\sigma}^2\right] $$

where *μ* and *σ* are location and scale parameters, respectively, and $\overline{\mathrm{y}}$ and s^2^ are sample mean and variance, respectively, of the log-transformed TRP concentration data y (Gelman et al. 2013). Uniform distributions in the ranges (−∞, +∞) and (0, +∞) were used as prior probability distributions for μ and σ, respectively.

The joint posterior distribution π(μ, σ| y) was sampled by MCMC using the proportionality property π(θ| y) ∝ L(θ| y) π(θ) of Bayes rule. One thousand realisations were generated after 1000 burn-in samples through Hamiltonian Monte Carlo using the Stan software in the R environment (R Core Team 2017; Stan Development Team 2017).

For each realisation of parameters *μ* and *σ*, the arithmetic mean of TRP was calculated as *m*_a_ = exp (μ + σ^2^/2), resulting in the posterior distribution of m_a_. The posterior distribution of the 95%ile of TRP was calculated accordingly using the quantile function for the lognormal distribution (Johnson et al. 1995).

### Non-parametric estimation with Bayesian bootstrap

The Bayesian bootstrap used in this study followed the method described in Aitkin (2010). A grid *Y* = {*Y*_1_, *Y*_2_, …, *Y*_J_, …, *Y*_D_} was defined over the range of feasible values of TRP with equidistant bins. The bin size was set equal to the detection limit (δ) of TRP (Krueger 2017). With this setup, the probability that the variable is located in the Jth bin *Y*_J_ is *p*_J_ = N_J_/∑_J_N_J_, the proportion of the population count *N*_J_ in *Y*_J_. The maximum value of the grid *Y*_D_ was defined as 1.5 times the maximum value of the high-frequency data; Y_1_ was defined as 0 mg P L^−1^ and δ as 0.003 mg P L^−1^, the detection limit (Cassidy and Jordan 2011).

The likelihood function of the multinomial distribution given sample counts n_J_ is

9 $$ \mathrm{L}\left({\mathrm{p}}_1,{\mathrm{p}}_2,\cdots, {\mathrm{p}}_{\mathrm{D}}|\mathrm{y}\right)=\left[\mathrm{n}!/{\prod}_{\mathrm{J}=1}^{\mathrm{D}}{\mathrm{n}}_{\mathrm{J}}!\right]\cdotp {\prod}_{\mathrm{J}=1}^{\mathrm{D}}{\mathrm{p}}_{\mathrm{J}}^{{\mathrm{n}}_{\mathrm{J}}} $$

The Dirichlet distribution is a natural conjugate to the multinomial distribution and assigns prior belief of how many data points are contained in a bin Y_J_ via parameter α_J_.

10 $$ \uppi \left({\mathrm{p}}_1,{\mathrm{p}}_2,\cdots, {\mathrm{p}}_{\mathrm{D}}\right)=\left[\Gamma \left({\sum}_{\mathrm{J}=1}^{\mathrm{D}}{\upalpha}_{\mathrm{J}}\right)/{\prod}_{\mathrm{J}=1}^{\mathrm{D}}\Gamma \left({\upalpha}_{\mathrm{J}}\right)\right]\cdotp {\prod}_{\mathrm{J}=1}^{\mathrm{D}}{\mathrm{p}}_{\mathrm{J}}^{\upalpha_{\mathrm{J}}-1} $$

The total sum of α_J_ assigned over the grid Y is $\upalpha \equiv {\sum}_{\mathrm{J}=1}^{\mathrm{D}}{\upalpha}_{\mathrm{J}}$, which represents the total weight of the assigned prior information. In this study, because of ambiguity in choosing α_J_ (Krueger 2017), the Haldane prior distribution with *α* = 0 was used, i.e. neglecting any unobserved bins.

The posterior distribution of p_J_ as product of the likelihood and the prior represents the probabilities of proportions of the population falling into certain bins given the observed data y and the prior assumptions.

11 $$ \uppi \left({\mathrm{p}}_1,{\mathrm{p}}_2,\cdots, {\mathrm{p}}_{\mathrm{D}}|\mathrm{y}\right)\propto \left[\Gamma \left({\sum}_{\mathrm{J}=1}^{\mathrm{D}}{\mathrm{n}}_{\mathrm{J}}+{\mathrm{a}}_{\mathrm{J}}\right)/{\prod}_{\mathrm{J}=1}^{\mathrm{D}}\Gamma \left({\mathrm{n}}_{\mathrm{J}}+{\mathrm{a}}_{\mathrm{J}}\right)\right]\cdotp {\prod}_{\mathrm{J}=1}^{\mathrm{D}}{\mathrm{p}}_{\mathrm{J}}^{{\mathrm{n}}_{\mathrm{J}}+{\mathrm{a}}_{\mathrm{J}}-1} $$

The posterior distribution of p_J_ was sampled by 1000 realisations using the R package MCMCpack (Martin et al. 2011).

As for the lognormal model, from each realisation of π(p_1_, p_2_, ⋯, p_D_| y), the arithmetic mean of the TRP concentration was calculated as ${m}_{\mathrm{a}}={\sum}_{\mathrm{J}=1}^{\mathrm{D}}{\mathrm{p}}_{\mathrm{J}}{\mathrm{Y}}_{\mathrm{J}}$, yielding the posterior distribution of m_a_. Accordingly, the posterior of the 95%ile was calculated as the largest value of the empirical cumulative distribution function that was smaller than or equal to 0.95.

## Abbreviations

BOD: Biochemical oxygen demand
DO: Dissolved oxygen
EU: European Union
HDI_95_: 95% highest density interval
OM: Operational monitoring
PDF: Probability density function
RMBE: Relative mean bias error
SM: Surveillance monitoring
TRP: Total reactive phosphorus
WFD: Water Framework Directive
